# Genome Sequence of Grapevine Virus T, a Novel Foveavirus Infecting Grapevine

**DOI:** 10.1128/genomeA.00995-17

**Published:** 2017-09-14

**Authors:** Yeonhwa Jo, Myung-Kyu Song, Hoseong Choi, Jae-Seong Park, Jae-Wung Lee, Sen Lian, Bong Choon Lee, Won Kyong Cho

**Affiliations:** a Department of Agricultural Biotechnology, College of Agriculture and Life Sciences, Seoul National University, Seoul, Republic of Korea; b Grape Research Institute, Chungbuk Agricultural Research and Extension Services, Okcheon, Republic of Korea; c College of Crop Protection and Agronomy, Qingdao Agricultural University, Qingdao, Shandong, China; d Crop Foundation Division, National Institute of Crop Science, RDA, Wanju, Republic of Korea

## Abstract

Here, we report the genome sequence of grapevine virus T (GVT), a novel single-stranded RNA virus identified from a transcriptome of grapevine. The genome of GVT is 8,701 nucleotides in length and encodes five open reading frames. GVT is a putative member of the genus *Foveavirus* in the family *Betaflexiviridae*.

## GENOME ANNOUNCEMENT

Grapevines (*Vitis vinifera*) are commercially important fruit crops worldwide. Grapevines are cultivated for the production of wine or table grapes. A wide range of pathogens, including viruses, can threaten the quality and quantity of grapevine production. To date, more than 70 plant viruses infecting grapevines have been reported (1).

With the advancement of next-generation sequencing (NGS) techniques, numerous plant viruses, including novel viruses, have been identified (2, 3). NGS techniques facilitate not only the identification of known and novel viruses but also the assembly of viral genomes (4, 5). For example, eight viruses and two viroids have been identified from the transcriptome data of a single grapevine cultivar (6).

In the search for viruses and viroids infecting grapevine using transcriptome data, a novel virus has been identified from a transcriptome of the grapevine cultivar Teroldego (7). Detailed information for the plant sample and library preparation can be found in the previous study (7). To summarize, the total mRNA was extracted by pooling three individual grape berry samples from a single cultivar. The library, prepared using the Illumina TruSeq RNA kit was single-end (85 bp) sequenced using the Illumina GAIIx platform. We downloaded the raw data (2.5 Gb) for grapevine cultivar Teroldego deposited in the Sequence Read Archive (SRA) database (GenBank accession number ERR923264). The raw sequence reads were *de novo* assembled with Trinity version 2.0.6 with default parameters (8). A BLAST search of the assembled transcriptome of Teroldego was completed against the NCBI viral reference database (https://www.ncbi.nlm.nih.gov/genome/viruses).

From the transcriptome of Teroldego, we identified 86 contigs associated with viruses and viroids. Of them, two contigs were associated with a novel virus named grapevine virus T (GVT) isolate Cho (GenBank accession number MF095096). The genome of GVT is composed of positive single-stranded RNA with a length of 8,701 nucleotides (nt). The obtained GVT genome sequence is a nearly full-length genome containing sequences of the 5′ and 3′ untranslated regions (61 and 246 nt in length, respectively). The GVT encodes five open reading frames (ORFs), replicase (ORF1), three triple-gene blocks (TGB1, TGB2, and TGB3), and coat protein (ORF5). The BLASTp search against NCBI’s nonredundant protein database revealed that the five ORFs of GVT shared the following sequence similarities with grapevine rupestris stem pitting-associated virus (GRSPaV): 52% identity with replicase, 62% identity with TGB1, 53% identity with TGB2, 48% identity with TGB3, and 53% identity with coat protein. Based on the BLASTp results, we proposed that the identified GVT is a putative member of the genus *Foveavirus* in the family *Betaflexiviridae*. Taken together, we report a novel virus, GVT, which is a potential member of the genus *Foveavirus*, from the grapevine transcriptome.

### Accession number(s).

The full-genome sequence of grapevine virus T isolate Cho has been submitted to GenBank under the accession number MF095096.
